# Timing optimization of low-dose first-pass analysis dynamic CT myocardial perfusion measurement: validation in a swine model

**DOI:** 10.1186/s41747-019-0093-6

**Published:** 2019-04-03

**Authors:** Logan Hubbard, Shant Malkasian, Yixiao Zhao, Pablo Abbona, Sabee Molloi

**Affiliations:** Department of Radiological Sciences, Medical Sciences I, B-140, University of California, Irvine, Irvine, CA 92697 USA

**Keywords:** Animal experimentation, Coronary artery disease, Myocardium, Perfusion imaging, Tomography (x-ray computed)

## Abstract

**Background:**

Myocardial perfusion measurement with a low-dose first-pass analysis (FPA) dynamic computed tomography (CT) perfusion technique depends upon acquisition of two whole-heart volume scans at the base and peak of the aortic enhancement. Hence, the objective of this study was to validate an optimal timing protocol for volume scan acquisition at the base and peak of the aortic enhancement.

**Methods:**

Contrast-enhanced CT of 28 Yorkshire swine (weight, 55 ± 24 kg, mean ± standard deviation) was performed under rest and stress conditions over 20–30 s to capture the aortic enhancement curves. From these curves, an optimal timing protocol was simulated, where one volume scan was acquired at the base of the aortic enhancement while a second volume scan was acquired at the peak of the aortic enhancement. Low-dose FPA perfusion measurements (*P*_FPA_) were then derived and quantitatively compared to the previously validated retrospective FPA perfusion measurements as a reference standard (*P*_REF_). The 32-cm diameter volume CT dose index, ${\mathrm{CTDI}}_{\mathrm{vol}}^{32}$ and size-specific dose estimate (SSDE) of the low-dose FPA perfusion protocol were also determined.

**Results:**

*P*_FPA_ were related to the reference standard by *P*_FPA_ = 0.95 · *P*_REF_ + 0.07 (*r* = 0.94, root-mean-square error = 0.27 mL/min/g, root-mean-square deviation = 0.04 mL/min/g). The ${\mathrm{CTDI}}_{\mathrm{vol}}^{32}$ and SSDE of the low-dose FPA perfusion protocol were 9.2 mGy and 14.6 mGy, respectively.

**Conclusions:**

An optimal timing protocol for volume scan acquisition at the base and peak of the aortic enhancement was retrospectively validated and has the potential to be used to implement an accurate, low-dose, FPA perfusion technique.

## Key points

- A new first-pass analysis dynamic CT perfusion technique enables accurate myocardial perfusion measurement at a low radiation dose, but its clinical feasibility depends upon optimal acquisition timing.
- Optimal acquisition timing can be determined using the contrast bolus injection time alone, as a robust mathematical relation exists between the contrast bolus injection time and the time-to-peak aortic enhancement, independent of heart rate or maximal peak enhancement.

## Background

Accurate physiological assessment of coronary artery disease (CAD) is necessary for objective definition of patient risk [1–4]. Such physiological assessment of risk is feasible with dynamic computed tomography (CT) perfusion [3, 5, 6], where the spatial distribution of absolute stress myocardial perfusion in mL/min/g, in combination with physiological cutoff thresholds, can be used to reliably stratify patient risk and properly guide intervention [7, 8]. Nevertheless, current dynamic CT perfusion techniques are known to be inaccurate [9–11] and have limited craniocaudal coverage. Such techniques also deliver high effective radiation doses from 5.3 to 10 mSv per stress or rest perfusion measurement, despite attempts to reduce tube voltage, modulate tube current, and reduce the number of acquisitions [12–15].

Fortunately, a new first-pass analysis (FPA) dynamic CT perfusion technique has been shown to improve the accuracy of perfusion measurement through whole-heart craniocaudal coverage, with additional potential to reduce the effective radiation dose through maximal acquisition number reduction. More specifically, the FPA perfusion technique is enabled by 320-slice CT scanner technology that allows the entire myocardium to be acquired in a single gantry rotation. As such, the technique only requires two optimally timed first-pass volume scans acquired at the base and the peak of the aortic enhancement for accurate perfusion measurement [16–18]. Moreover, given such a sampling scheme, if using a combined rest and stress perfusion protocol, the second volume scan of the rest protocol may also be used for CT angiography if acquired at a diagnostic tube current [16–18]. While proper timing of the two first-pass volume scans may be determined through the preemptive use of a diluted test bolus [19, 20], extra contrast and radiation dose are required. Alternately, dynamic bolus tracking-based prospective timing and acquisition of the two first-pass volume scans may provide a better solution but remains to be developed and optimized.

Hence, the objective of this study was to retrospectively develop a robust mathematical relation between the contrast bolus injection time and contrast bolus time-to-peak for proper prospective acquisition of the two first-pass volume scans. The central hypothesis was that the ideal delay time between the two volume scans equals one half the contrast injection time plus a fixed dispersion delay. Based on the results, an optimal dynamic bolus tracking-based timing protocol was validated for the low-dose FPA perfusion technique.

## Methods

### First-pass analysis perfusion measurement theory

**Fig. 1:**
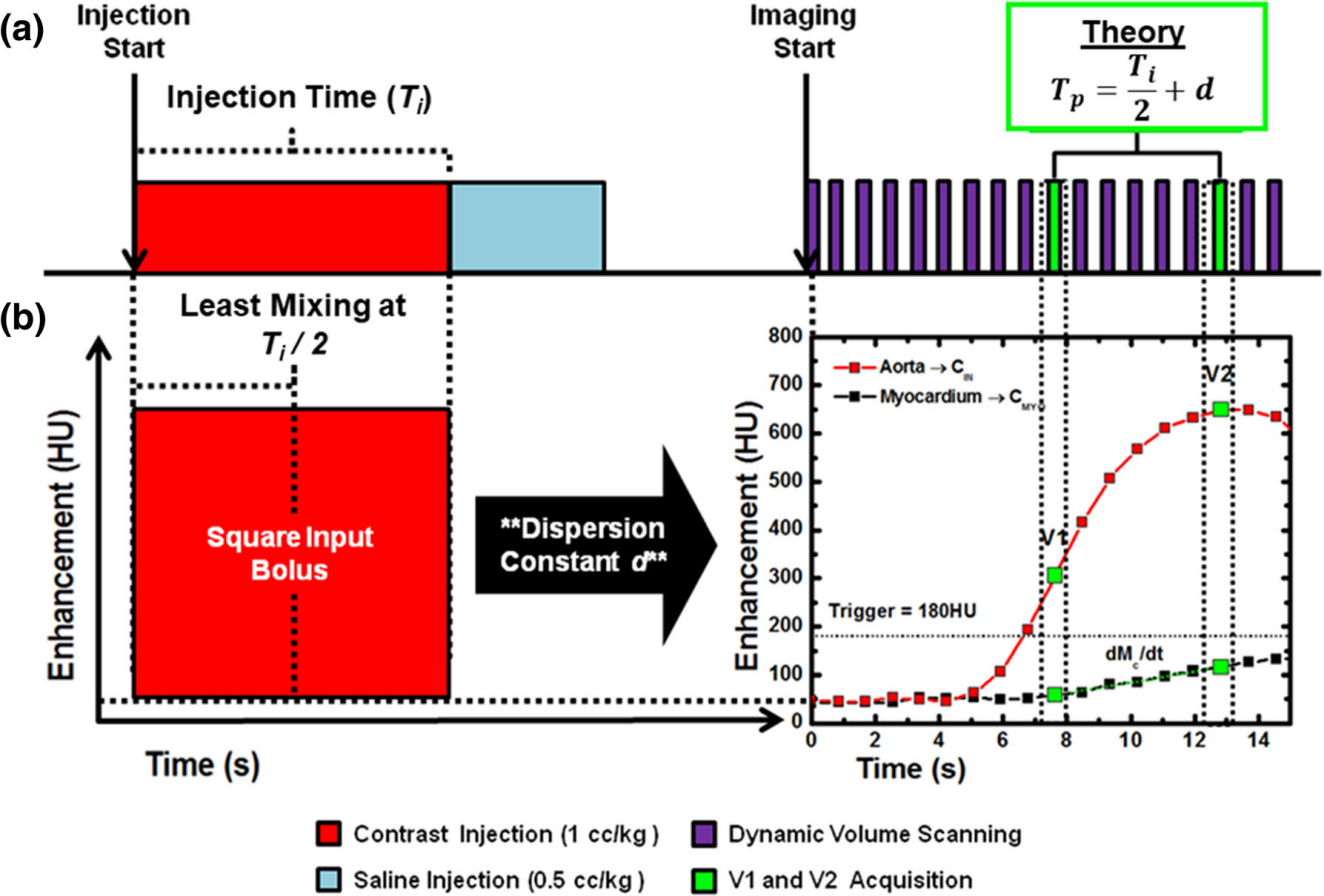
Contrast bolus injection protocol and bolus dispersion theory. **a** For all contrast-enhanced imaging, contrast was injected at a fixed rate, followed by a saline chaser at the same rate. Dynamic imaging of the heart was then performed. **b** The contrast bolus dispersion and optimal peak timing theory states that the peak enhancement of the contrast bolus in the aorta occurs at approximately one half the injection time plus a fixed dispersion delay ($\frac{T_{\mathrm{i}}}{2}+\mathrm{d}$). Note that the V2 volume scan also corresponds to the site of optimal CT angiography

The FPA perfusion technique is enabled by 320-slice CT scanner technology and models the entire myocardium as a single lumped compartment. Given this model [21], the average perfusion within the compartment is proportional to the first-pass entry of contrast material mass over time (dM_C_/dt), normalized by the incoming contrast material concentration (*C*_in_) and compartment tissue mass (*M*_*T*_), assuming no contrast material exits over the measurement duration. By extension, only two optimally timed first-pass volume scans, labeled V1 and V2 in Fig. 1, are mathematically necessary for FPA perfusion measurement, as previously validated versus invasive fractional flow reserve, quantitative microsphere perfusion, and ultrasonic flow probe measurement [16–18]. V1 is defined as a volume scan at approximately the base of the aortic enhancement, while V2 is defined as a volume scan at approximately the peak of aortic enhancement, *i.e.*, at the same point necessary for optimal CT angiography [20]. The integrated change in myocardial enhancement (dM_C_/dt), the average change in myocardial enhancement (ΔHU_AVE_), the average aortic blood pool enhancement (*C*_in_), and the voxel-by-voxel change in myocardial enhancement (ΔHU) between the V1 and V2 volume scans may then be used in combination with the total myocardial mass (*M*_*T*_) to derive voxel-by-voxel perfusion (*P*_FPA_) [16–18], as described by Eq. 1:

1 $$ {P}_{\mathrm{FPA}}={\left({M}_T^{-1}{C}_{\mathrm{in}}^{-1}\frac{{\mathrm{dM}}_{\mathrm{c}}}{\mathrm{dt}}\right)}_{\mathrm{AVE}}\cdot \frac{\varDelta \mathrm{HU}}{\varDelta {\mathrm{HU}}_{\mathrm{AVE}}} $$

### Optimal peak timing theory

Garcia et al. [22] and Han et al. [23] have shown that the temporal width of the contrast bolus in the aorta is proportional to the volume of the contrast bolus for a given injection rate. By extension, it follows that for any injection rate, the contrast bolus injection time should also be predictive of the temporal width of the contrast bolus in the aorta.

Specifically, upon injection, the contrast bolus is approximately square in shape, *i.e.*, it has a fixed concentration and enhancement per unit time, as displayed in Fig. 1a, b. However, the pulsatile nature of the circulatory system cause mixing and dispersion, resulting in a broad, approximately gamma variate contrast bolus geometry by the time it arrives in the aorta, as displayed in Fig. 1b. Nevertheless, such mixing and dispersion primarily impact the leading and trailing edges of the bolus. Hence, the temporal center of the bolus maintains the highest contrast concentration and peak enhancement where one half the injection time ($\frac{T_i}{2}$) plus a fixed dispersion delay (*T*_*d*_) should approximately correspond to the temporal center of the contrast bolus in the aorta (*T*_*p*_), as described by Eq. 2 and displayed in Fig. 1b:

2 $$ {T}_p=\frac{T_{\boldsymbol{i}}}{2}+{T}_d $$

### General methods

The study was approved by the Institutional Animal Care and Use Committee. Contrast-enhanced dynamic CT imaging was performed under rest and stress conditions in 28 Yorkshire swine (weight 55 ± 24 kg, mean ± standard deviation), with one to two rest acquisitions followed by one to three stress acquisitions per animal, all with the same CT settings. Five of the swine also had significant coronary artery disease generated with a balloon stenosis method as previously described [16–18]. All data was prospectively acquired and retrospectively analyzed. First, the contrast bolus geometry in the aorta was characterized using automatic gamma variate fitting. From the fit curves, the ideal time-to-peak enhancement between the base and peak of the aortic enhancement was derived and compared to one half the contrast injection time. Next, the ideal time-to-peak enhancement was used to design a practical, low-dose FPA perfusion protocol, where the first volume scan (V1) was acquired at approximately the base of the aortic enhancement through dynamic bolus tracking with triggering at 180 HU, while the second volume scan (V2) was acquired *T*_*p*_ seconds after V1, at approximately the peak of the aortic enhancement. More specifically, *T*_*d*_ was incrementally increased from 0 to 4 s and the accuracy of peak acquisition was assessed in each case, with the best *T*_*d*_ selected for the low-dose FPA perfusion protocol. Finally, the accuracy of perfusion measurement with the low-dose FPA perfusion protocol was assessed in a subset of 14 animals, where low-dose FPA perfusion measurements were quantitatively compared to previously validated reference standard retrospective FPA perfusion measurements from the same 14 animals [16–18].

### Animal preparation

Anesthesia was induced with Telazol (4.4 mg/kg), ketamine (2.2 mg/kg), and xylazine (2.2 mg/kg), and was maintained with 1.5–2.5% isoflurane (Highland Medical Equipment, Temecula, CA and Baxter, Deerfield, IL, USA). Sheaths were placed (5Fr, AVANTI®, Cordis Corporation, Miami Lakes, FL, USA) in each femoral vein and were used for fluid, adenosine, and contrast material intravenous administration.

### Contrast-enhanced imaging protocol

For each animal, one or two rest acquisitions were performed, followed by one to three stress acquisitions, all with the same CT settings. For each stress acquisition, adenosine was infused intravenously for 3 min prior to and throughout imaging (240 μg adenosine/kg/min, Model 55-2222, Harvard Apparatus, Holliston, MA, USA). For each rest and stress acquisition, 1 mL/kg of contrast material (Isovue 370, Bracco Diagnostics, Princeton, NJ, USA) was injected (5 mL/s, Empower CTA, Acist Medical Systems, Eden Prairie, MN, USA) followed by a saline chaser (0.5 mL/kg) at the same rate. ECG-gated volume scans were acquired dynamically with a 320-slice CT scanner (Aquilion One, Toshiba America Medical Systems, Tustin, CA, USA) over 20–30 s to capture base and peak of the aortic enhancement, as shown in Fig. 1a, b. All volume scans were acquired as full projection data at 100 kVp and 200 mA with a rotation time of 0.35 s, a collimation of 320 × 0.5 mm, and a cranio-caudal coverage of 16 cm. A minimum 10-min delay was employed between consecutive acquisitions to allow for adequate clearance of contrast material and, if used, adenosine. All volume scans were retrospectively reconstructed at 75% of the R-R interval using an adaptive iterative dose reduction three-dimensional reconstruction [24], an FC03 kernel, and a voxel size of 0.43 × 0.43 × 0.5 mm.

### Contrast bolus parameter assessment

**Fig. 2:**
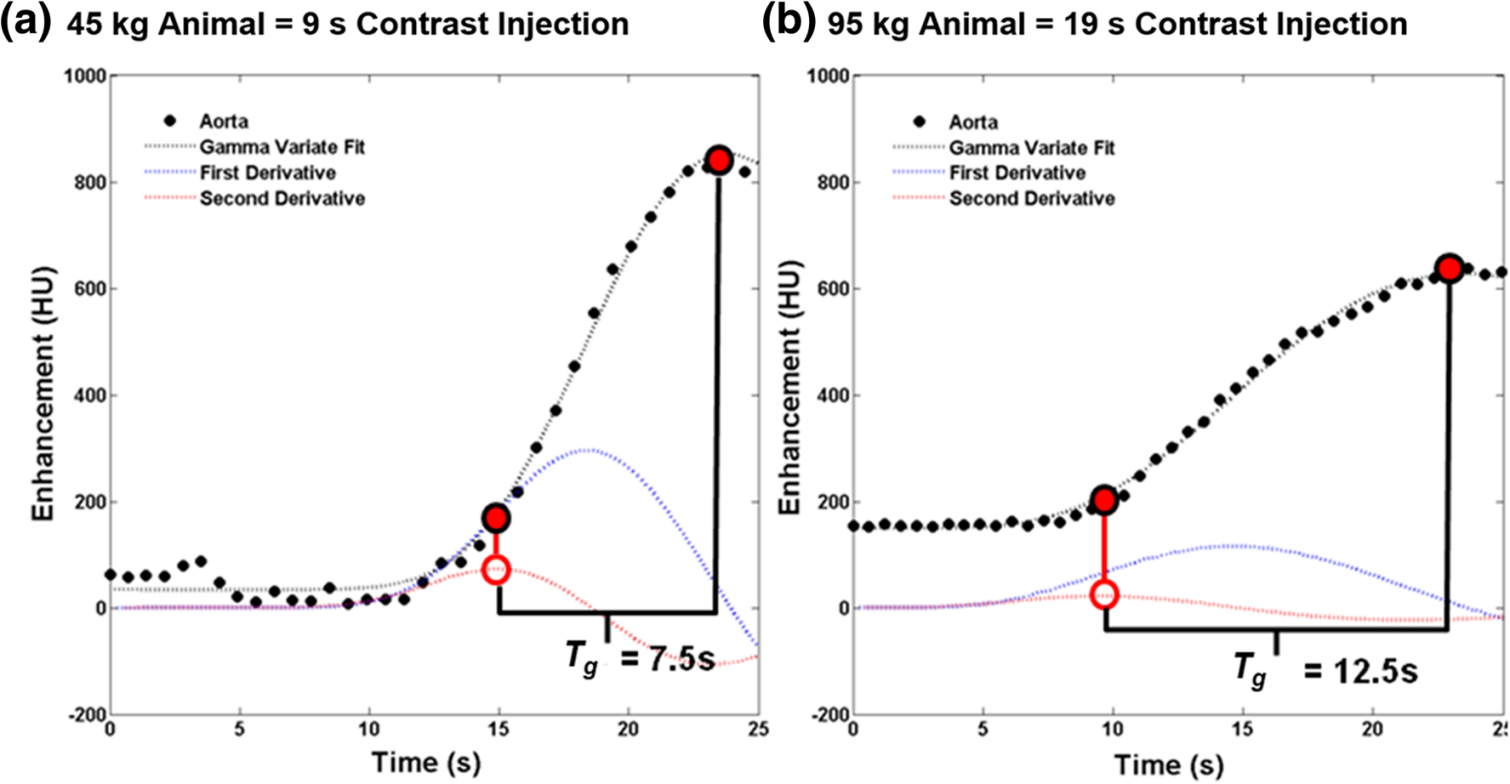
Automatic gamma variate fitting of the aortic enhancement in two swine. **a** The gamma fit (black), first derivative (blue), second derivative (red), and time-to-peak (*T*_*g*_) are displayed for a 45-kg animal with a 9-s contrast injection. **b** The gamma fit (black), first derivative (blue), second derivative (red), and time-to-peak (*T*_*g*_) are also displayed for a 95-kg animal with a 19-s contrast injection

For each acquisition, the central lumen of the aorta was segmented semi-automatically (Vitrea fX version 6.0, Vital Images, Inc., Minnetonka, MN, USA), yielding a vascular volume of interest. The volumes of interests were then used to generate aortic enhancement curves that were automatically fit via least squares fitting (LSQCurveFit, MatLab 2013a, MathWorks, Natick, MA, USA) using a gamma variate function of the form:

3 $$ \mathrm{Enhancement}(t)=A{\left(\frac{t}{\tau}\right)}^b\cdot {\exp}^{b\left(\frac{1-t}{\tau}\right)}+C $$

where *A* is the maximum aortic enhancement, *t* is time, *τ* is the decay constant, *b* is the power, and *C* is the initial pre-contrast aortic enhancement. The first and second derivatives of the gamma fit were also computed. The ideal time-to-peak (*T*_*g*_) in seconds between the ideal base and ideal peak of the aortic enhancement was calculated as the time difference between the maximum of the second derivative of the gamma variate fit and the peak of the actual gamma variate fit. The results were then compared to one half the contrast injection time using regression analysis. Example gamma fits are shown in Fig. 2. The gamma variate fit results were also analyzed to determine if there were pair-wise differences in heart rate, time-to-peak, and peak enhancement between rest and stress conditions. Additionally, in the five animals with significant stenoses, the gamma variate fit results were further analyzed to determine if there were pair-wise differences in time-to-peak between rest or stress acquisitions without stenosis as compared to stress acquisitions with significant stenosis.

### Practical prospective acquisition of the V1 and V2 scans

The optimal peak timing theory was then used to design a practical low-dose FPA perfusion protocol. First, dynamic bolus tracking with triggering at 140 HU above the baseline blood pool intensity in the aorta was simulated (SureStart, Aquilion One, Canon Medical Systems, Tustin, CA, USA), followed by systematic selection of V1 as the first electrocardiographically gated volume scan after triggering, corresponding to approximately the base of the aortic enhancement. Next, the delay time between V1 and V2 for acquisition of V2 at approximately the peak of the aortic enhancement was determined. V2 was systematically selected as the first ECG-gated volume scan $\frac{T_i}{2}+d$ s after V1, where the dispersion delay *d* was iteratively increased from 0.0 to 4.0 s. For each iteration, the temporal difference between V2 and the ideal peak was recorded in units of cardiac cycles. The results were quantitatively compared to determine the dispersion delay necessary for optimal prospective acquisition of V2 at approximately the peak of the aortic enhancement.

### Accuracy of prospective perfusion measurement

The accuracy of the low-dose FPA perfusion protocol was assessed in a subset of fourteen animals using previously validated retrospective FPA perfusion measurements as the reference standard. For each acquisition, V1 and V2 were systematically selected using the simulated acquisition protocol. Both volume scans were registered and combined into a maximum intensity projection image volume, which was segmented semi-automatically (Vitrea fX version 6.0, Vital Images, Inc., Minnetonka, MN, USA), yielding the entire myocardium. Low-dose FPA perfusion measurements were then computed according to Eq. 1 and were compared to the previously validated reference standard retrospective FPA perfusion measurements from the same fourteen animals [16–18]. Additionally, the 32-cm diameter volume CT dose index, ${\mathrm{CTDI}}_{\mathrm{vol}}^{32}$, of the low-dose FPA perfusion protocol was recorded, and a size-specific dose estimate (SSDE) was computed to account for the small 23-cm effective diameter of the swine [25].

### Statistical analysis

First, the gamma variate time-to-peak data was related to the injection time data through regression, root-mean-square error (RMSE), and root-mean-square deviation (RMSD) analysis, where RMSE and RMSD were defined as the square root of the standard deviation of the identity line and fit line residuals, respectively. Next, the rest and stress data, stenosis data, as well as the optimal V2 acquisition data, were assessed through paired sample *t* tests. Finally, all low-dose FPA perfusion measurements were quantitatively compared to the reference standard retrospective FPA perfusion measurements using regression, Bland-Altman, root-mean-square error, root-mean-square deviation, Lin’s concordance correlation coefficient [26], and paired sample *t* testing. Values of *p* lower than 0.050 were considered as significant. Statistical software was used for all analysis (MatLab 2013a, MathWorks, Natick, MA, USA; PS, Version 3.0, Vanderbilt University, Nashville, TN, USA; SPSS, Version 22, IBM Corporation, Armonk, NY, USA).

## Results

### General

A total of 98 measurements were obtained from the 28 animals used in the study. The weight of the animals was 52.2 ± 14.6 kg (mean ± standard deviation). The average rest and stress heart rates were 84.86 ± 13.67 and 93.04 ± 12.09 beats per min, respectively.

### Contrast bolus parameter assessment

**Fig. 3:**
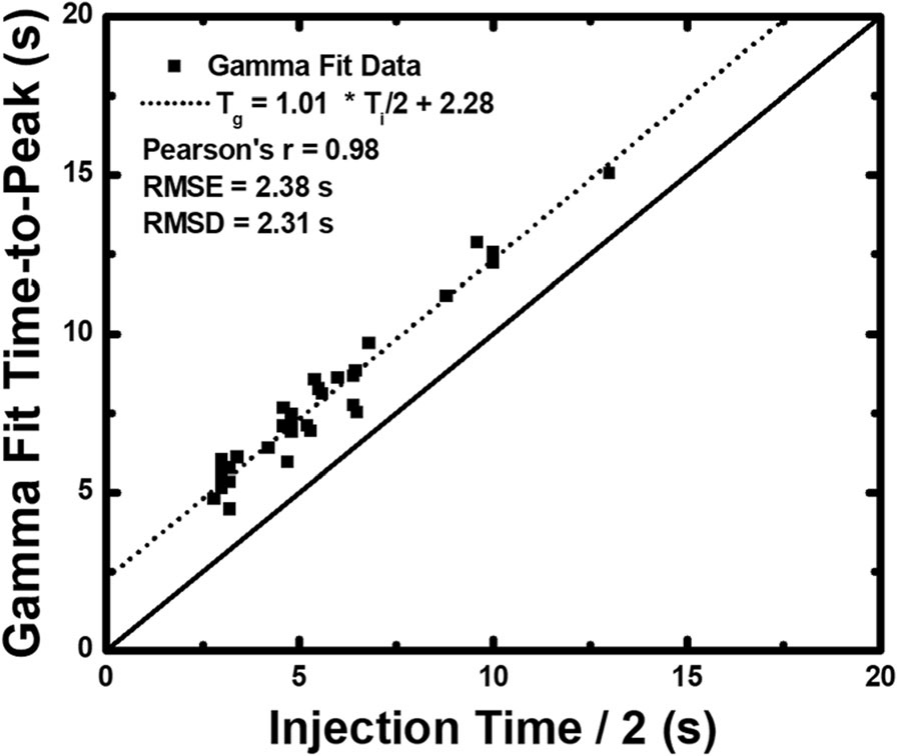
Gamma fit time-to-peak data as compared to the one half injection time data. A total of 98 measurements from 28 animals were obtained, and the time-to-peak of the aortic enhancement in each case was retrospectively assessed through automatic gamma variate fitting. The average gamma fit time-to-peak data from each animal was then quantitatively compared to the ideal bolus timing theory through regression analysis. *T*_*g*_ time-to-peak of the aortic enhancement, defined as the time between the peak of the second derivative of the gamma fit and the true peak of the gamma fit, *T*_*i*_ contrast injection time, RMSD root-mean-square deviation, RMSE root-mean-square error

The average gamma fit time-to-peak (*T*_*g*_) data from each animal was used for regression analysis. The gamma fit time-to-peak (*T*_*g*_) data and one half the injection time data ($\frac{T_i}{2}$) were related by *T*_*g*_ = 1.01  · $\frac{T_i}{2}$+  2.28 s, with a Pearson’s correlation of *r* = 0.98, a root-mean-square error of 2.28 s, and a root-mean-square deviation of 2.31 s, as shown in Fig. 3. For all the animals, the average heart rate, time-to-peak, and peak enhancement under rest and stress conditions with paired sample *t* test comparisons are shown in Table 1. For the five animals with significant stenosis, the average time-to-peak for rest or stress acquisitions without stenosis was 7.29 ± 0.82 s, while the average time-to-peak for stress acquisitions in the presence of significant stenosis was 7.10 ± 0.40 s (*p* = 0.473).

**Table 1:** Rest and stress comparison

Parameter (*n* = 28)	Rest (mean ± SD)	Stress (mean ± SD)	*p* value
Heart rate (beats per min)	84.86 ± 13.67	93.04 ± 12.09	0.007
Gamma fit time-to-peak (s)	7.41 ± 1.93	7.19 ± 1.95	0.411
Peak enhancement (HU)	640.07 ± 150.12	540.07 ± 149.24	0.012

*n* number of animals assessed, *SD* standard deviation

### Optimal prospective acquisition of the V1 and V2 scans

**Fig. 4:**
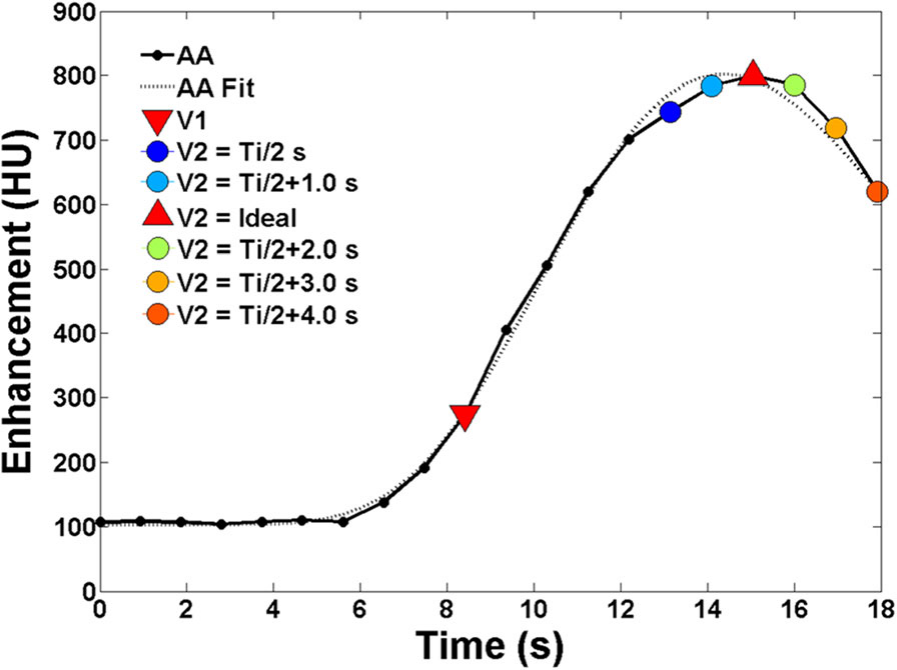
Low-dose FPA perfusion protocol simulation. The aortic gamma variate fit was used to simulate dynamic bolus tracking-based triggering, where V1 was systematically selected as the first electrocardiographically gated volume scan after triggering, while V2 was systematically selected as the first electrocardiographically gated volume scan occurring $\frac{T_{\mathrm{i}}}{2}$+ 0 to 4.0 s after V1. The cardiac cycle distance of V2 from the ideal peak enhancement (red triangle) was then recorded. Note that the V2 volume scan also corresponds to the site of optimal CT angiography

A total of 98 measurements from 28 animals were assessed. Data from the three best dispersion delays “*d*” in seconds is shown in Table 2. Optimal time delays of $\frac{T_i}{2}$+ 0.5, $\frac{T_i}{2}$+ 1, and $\frac{T_i}{2}$+ 1.5 s resulted in an average cardiac cycle difference of -0.56 ± 1.79, 0.13 ± 1.85, and 0.86 ± 1.97 (mean ± standard deviation) between V2 and the ideal peak, respectively. An example of the low-dose FPA perfusion protocol simulation is shown in Fig. 4.

**Table 2:** Low-dose peak acquisition simulation as compared to ideal peak acquisition

Protocol (*n* = 98)	Cardiac cycle difference from peak (beats, mean)	*p* value (comparison with the ideal peak)	RMSE (beats)
$\frac{{\mathrm{T}}_{\mathrm{i}}}{2}$+ 0.5 s	-0.56 ± 1.79	0.004	1.87
$\frac{{\mathrm{T}}_{\mathrm{i}}}{2}$ + 1.0 s	0.13 ± 1.85	0.497	1.84
$\frac{{\mathrm{T}}_{\mathrm{i}}}{2}$ + 1.5 s	0.86 ± 1.97	0.000	2.14

*n* number of measurements made in the 28 animals, $\frac{T_i}{2}$ one half of the contrast injection time in seconds, *RMSE* root-mean-square error

### Accuracy of prospective perfusion measurement

**Fig. 5:**
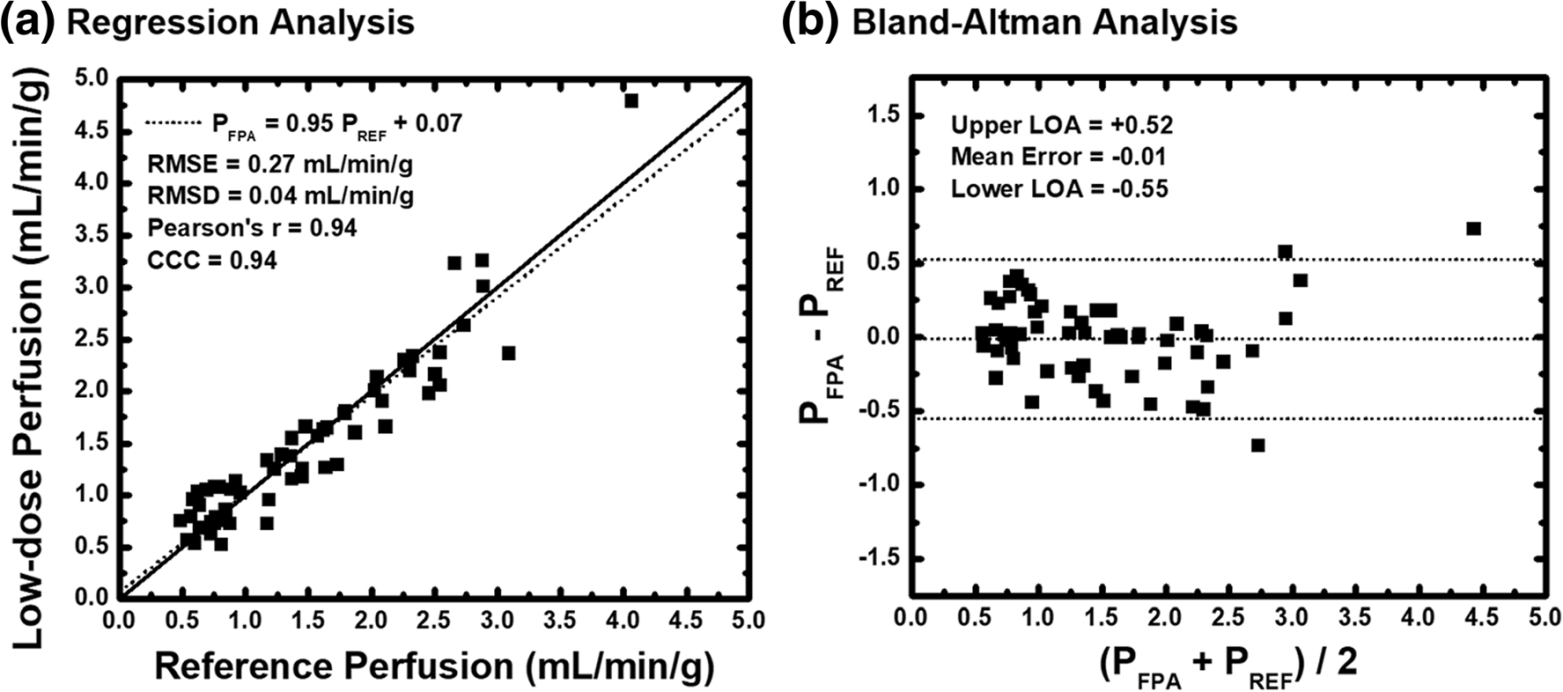
Low-dose first-pass analysis (FPA) perfusion measurements as compared to the reference standard retrospective FPA perfusion measurements. A total of 60 perfusion measurements from 14 animals were assessed. **a** Regression analysis comparing the simulated low-dose FPA perfusion measurements to the previously validated reference standard retrospective FPA perfusion measurements is displayed. **b** Corresponding Bland-Altman analysis is also shown

**Fig. 6:**
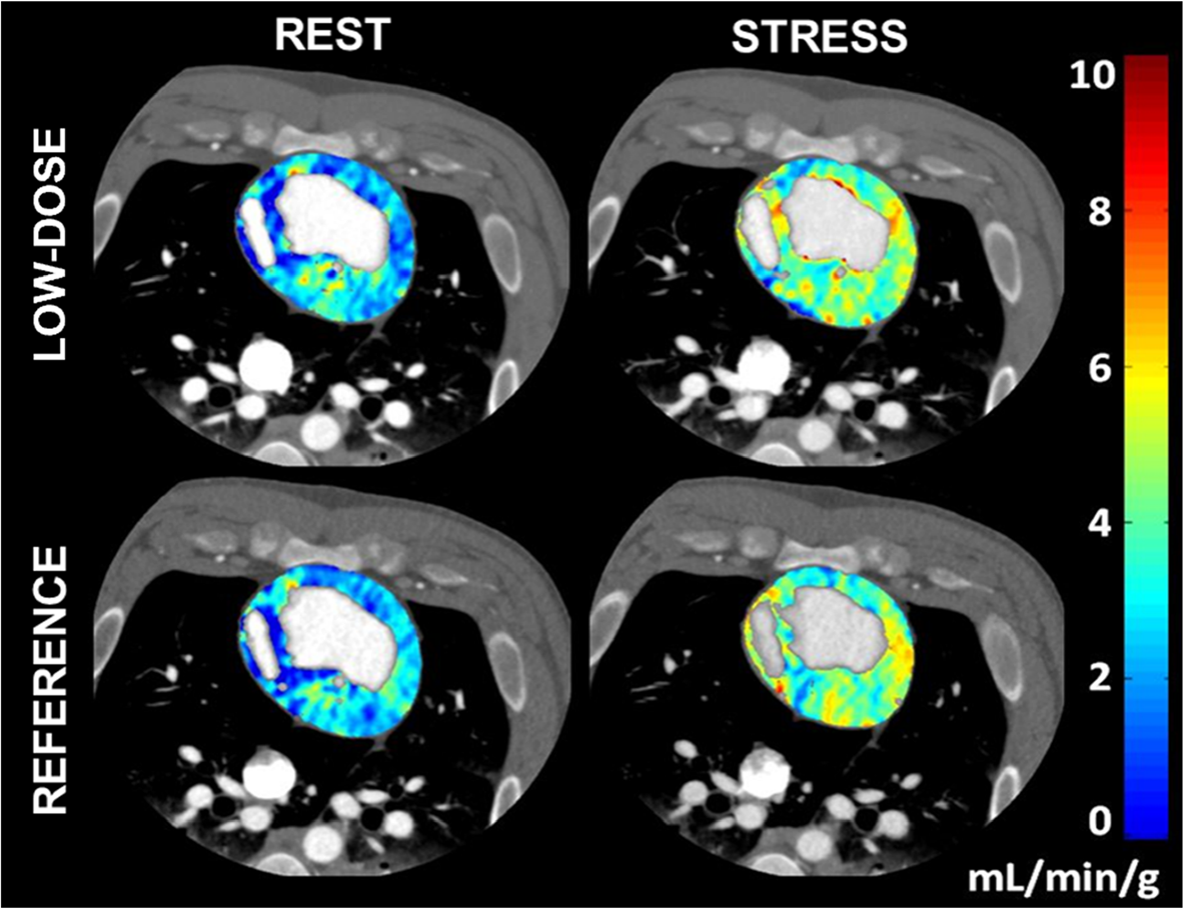
Quantitative voxel-by-voxel first-pass analysis (FPA) perfusion maps. Low-dose rest (top left) and stress (top right) FPA perfusion maps are displayed as compared to reference standard retrospective rest (bottom left) and stress (bottom right) FPA perfusion maps. The color bar indicates myocardial perfusion in mL/min/g.

A total of 60 perfusion measurements from 14 animals were assessed. Low-dose FPA perfusion measurements (*P*_FPA_) were generated using the best dispersion delay “*d* = 1 s” and were related to the previously validated reference standard FPA perfusion measurements (*P*_REF_) by *P*_FPA_ = 0.95 ·*P*_REF_ + 0.07 mL/min/g, with a Pearson’s correlation of *r* = 0.94, a concordance correlation of *ρ* = 0.94, a root-mean-square error of 0.27 mL/min/g, and a root-mean-square deviation of 0.04 mL/min/g, as shown in Fig. 5a**,** with corresponding Bland-Altman analysis displayed in Fig. 5b. Additionally, the mean of the low-dose measurements was 1.51 ± 0.80 mL/min/g, while the mean of the reference standard measurements was 1.49 ± 0.80 (*p* = 0.691). More importantly, the ${\mathrm{CTDI}}_{\mathrm{vol}}^{32}$ and SSDE of the low-dose FPA perfusion protocol were found to be 9.2 mGy and 14.6 mGy, respectively, corresponding to an effective dose and size-specific effective dose of 2.1 and 3.3 mSv, respectively, as estimated using the craniocaudal coverage of 16 cm and an adult chest conversion factor of *k* = 0.014. Perfusion maps were also generated to compare the spatial distribution of low-dose FPA perfusion measurements to the corresponding reference standard FPA perfusion measurements, as shown in Fig. 6.

## Discussion

The first goal of the study was to develop a robust mathematical relation between the contrast bolus injection time and contrast bolus time-to-peak. The results indicate that over a large range of heart rates (59.74–126.94 beats per min), injection volumes (28–96 mL), and injection times (5.6–19.2 s), one half the injection time plus a fixed dispersion delay of approximately 2.3 s ($\frac{T_i}{2}+2.3\ \mathrm{s}$) corresponds to the ideal time-to-peak aortic enhancement. Furthermore, in all the animals, there was no significant difference in the ideal time-to-peak enhancement between rest and stress conditions (*p* = 0.411), although stress peak enhancement was significantly lower than rest peak enhancement (*p* = 0.012) and stress heart rates were significantly higher than rest heart rates (*p* = 0.007). That said, such findings are in agreement with prior work by Bae et al., which describes an inverse relationship between cardiac output and peak enhancement [27]. More importantly, in the five animals with significant stenosis, there were no significant differences in the ideal time-to-peak enhancement between rest or stress conditions without stenosis as compared to stress conditions in the presence of significant stenosis (*p* = 0.473). Given these data, the contrast bolus injection time is strongly predictive of the ideal time-to-peak enhancement [22, 23], independent of heart rate, peak enhancement, or significant stenosis, *i.e.*, the time-to-peak enhancement can be predicted from the contrast injection protocol alone. Such findings have important implications. Not only do they inform that optimal timing for the new, low-dose FPA perfusion technique is feasible, they also indicate the potential for optimal timing for CT angiography, as they enable the peak of the aortic enhancement to always be acquired independent of injection protocol.

Using the above relation, the second goal of the study was to develop a practical acquisition protocol for the low-dose FPA perfusion technique. The results indicate that with dynamic bolus tracking, practical acquisition of V1 at approximately the base of the aortic enhancement is feasible, with only minor temporal delays. Moreover, a delay time of $\frac{T_i}{2}+1\ \mathrm{s}$ between V1 and V2 enabled practical acquisition of V2 at approximately the peak of the aortic enhancement, with no significant mean difference in cardiac cycle offset from the ideal peak (*p* = 0.497) and a RMSE of less than two cardiac cycles. Such findings inform that a low-dose FPA perfusion protocol, as well as optimal CT angiography, is feasible using dynamic bolus tracking with reliable peak acquisition timing. By extension, this work also has the potential to improve the diagnostic sensitivity of static perfusion measurement. More specifically, Pelgrim et al. [28] found that a fixed timed delay of approximately 8.3 ± 3.8 s between volume scan triggering and acquisition resulted in the highest contrast-to-noise ratio difference between normal and ischemic myocardium. That said, the deviation in optimal timing was large; hence, one half the injection time plus a fixed dispersion delay between volume scan triggering and acquisition could further improve the contrast-to-noise ratio and provide maximal diagnostic sensitivity, although prospective validation is still necessary.

Based on the above results, the final goal of the study was to validate the accuracy of the low-dose FPA perfusion protocol. Using a time delay of $\frac{T_i}{2}+1$ s between V1 and V2, low-dose FPA perfusion measurements were in good agreement with the previously validated reference standard retrospective FPA perfusion measurements, demonstrating near unity slope, minimal offset, good concordance correlation, and negligible bias, without significant differences between measurement means. Additionally, the low-dose FPA perfusion maps were in good agreement with the corresponding reference standard FPA perfusion maps. More importantly, such measurements were achieved at a ${\mathrm{CTDI}}_{\mathrm{vol}}^{32}$ and SSDE of only 9.2 mGy and 14.6 mGy, respectively, corresponding to an effective dose and size-specific effective dose of 2.1 and 3.3 mSv, substantially lower than the 5.3–10 mSv effective dose of current dynamic CT perfusion techniques. That said, other groups like van Assen et al. [29] have attempted to reduce the number of volume scans necessary for perfusion measurement. Unfortunately, given the mathematical limitations of most perfusion models combined with the limited craniocaudal coverage of standard CT scanner technology, lower temporal sampling rates invariably worsen the inaccuracies of current dynamic CT perfusion techniques [12–15, 29]. Fortunately, the low-dose FPA perfusion technique is fundamentally different from current dynamic CT perfusion techniques in that only two whole-heart volume scans are necessary for accurate perfusion measurement, as previously validated versus invasive fractional flow reserve (FFR), quantitative microsphere perfusion, and ultrasonic flow probe measurement [16–18]. More importantly, given the results of this work, practical implementation of the low-dose FPA perfusion technique may now be achieved.

This study has limitations. First, while a range of swine weights and injections times were used, the animals were undersized as compared to an average human cardiac patient. Hence, additional work in larger animals may still be necessary to confirm that the optimal timing protocol is robust. Second, all contrast injections were made at a fixed rate of 5 mL/s, *i.e.*, no other injection rates were assessed. Nevertheless, as the optimal timing protocol is predominantly a function of injection time, it should accommodate the use of any injection rate, although additional work may still be necessary to determine if a dispersion delay of 1 s remains valid for different injection rates. Third, only five swine in this study had balloon stenoses; hence, additional work in more swine with significant cardiac disease may still be necessary to better assess the performance of the optimal timing protocol. Fourth, the optimal timing protocol was developed and validated retrospectively using data from a wide-volume CT scanner, *i.e.*, the triggering scheme and subsequent timing of V1 and V2 acquisition may be scanner specific. As such, additional validation of the optimal timing protocol with other CT systems may still necessary to determine if the fixed dispersion delay of 1 s remains robust. Finally, while simulated low-dose FPA perfusion measurements performed well as compared to the previously validated reference standard retrospective FPA perfusion measurements [16–18], true prospective acquisition of V1 and V2 using the optimal timing protocol was not assessed; hence, additional prospective work in more animals may still be necessary. Alternatively, if prospective acquisition of V1 and V2 proves difficult, the timing of V1 and V2 acquisition may also be predicted through the preemptive use of a low-dose diluted test bolus acquisition [19, 20], although the overall contrast and radiation dose associated with low-dose FPA perfusion measurement will be increased.

In conclusion, an optimal timing protocol for accurate, low-dose, FPA dynamic CT perfusion measurement was retrospectively simulated and validated in 28 swine over a range of heart rates, injection volumes, and injection time intervals. Using dynamic bolus tracking, the protocol resulted in robust acquisition of V1 at approximately the base of the aortic enhancement, with robust acquisition of V2 $\frac{T_i}{2}+1\ \mathrm{s}$ after V1 at approximately the peak of the aortic enhancement. Such findings have important implications in that they enable practical, low-dose FPA perfusion measurement at a ${\mathrm{CTDI}}_{\mathrm{vol}}^{32}$ and SSDE of only 9.2 mGy and 14.6 mGy, respectively. In summary, the optimal timing protocol was retrospectively validated in 28 swine and has the potential to be used for practical implementation of a new, low-dose FPA perfusion technique.

## Abbreviations

CAD: Coronary artery disease
CT: Computed tomography
${\mathrm{CTDI}}_{\mathrm{vol}}^{32}$: 32-cm-diameter volume computed tomography dose index
FPA: First-pass analysis
RMSD: Root-mean-square deviation
RMSE: Root-mean-square error
SSDE: Size-specific dose estimate
V1: Volume scan 1
V2: Volume scan 2

## References

[1] Pijls NH, Fearon WF, Tonino PA (2010). Fractional flow reserve versus angiography for guiding percutaneous coronary intervention in patients with multivessel coronary artery disease: 2-year follow-up of the FAME (Fractional Flow Reserve Versus Angiography for Multivessel Evaluation) study. J Am Coll Cardiol.

[2] De Bruyne B, Pijls NH, Kalesan B (2012). Fractional flow reserve–guided PCI versus medical therapy in stable coronary disease. New Engl J Med.

[3] Chen MY, Rochitte CE, Arbab-Zadeh A (2017). Prognostic value of combined CT angiography and myocardial perfusion imaging versus invasive coronary angiography and nuclear stress perfusion imaging in the prediction of major adverse cardiovascular events: the CORE320 Multicenter Study. Radiology.

[4] Murthy VL, Naya M, Foster CR (2011). Improved cardiac risk assessment with noninvasive measures of coronary flow reserve. Circulation.

[5] Lubbers M, Coenen A, Kofflard M (2018). Comprehensive cardiac CT with myocardial perfusion imaging versus functional testing in suspected coronary artery disease. The multicenter, randomized CRESCENT-II trial. JACC Cardiovasc Imaging.

[6] Bamberg F, Hinkel R, Schwarz F (2012). Accuracy of dynamic computed tomography adenosine stress myocardial perfusion imaging in estimating myocardial blood flow at various degrees of coronary artery stenosis using a porcine animal model. Invest Radiol.

[7] Johnson NP, Gould KL, Di Carli MF, Taqueti VR (2016). Invasive FFR and noninvasive CFR in the evaluation of ischemia: what is the future?. J Am Coll Cardiol.

[8] Johnson NP, Gould KL (2012). Integrating noninvasive absolute flow, coronary flow reserve, and ischemic thresholds into a comprehensive map of physiological severity. JACC Cardiovasc Imaging.

[9] Bindschadler M, Modgil D, Branch KR, La Riviere PJ, Alessio AM (2014). Comparison of blood flow models and acquisitions for quantitative myocardial perfusion estimation from dynamic CT. Phys Med Biol.

[10] Ishida M, Kitagawa K, Ichihara T (2016). Underestimation of myocardial blood flow by dynamic perfusion CT: explanations by two-compartment model analysis and limited temporal sampling of dynamic CT. J Cardiovasc Comput Tomogr.

[11] Schwarz F, Hinkel R, Baloch E (2013). Myocardial CT perfusion imaging in a large animal model: comparison of dynamic versus single-phase acquisitions. JACC Cardiovasc Imaging.

[12] Ho KT, Chua KC, Klotz E, Panknin C (2010). Stress and rest dynamic myocardial perfusion imaging by evaluation of complete time-attenuation curves with dual-source CT. JACC Cardiovasc Imaging.

[13] Rossi A, Merkus D, Klotz E, Mollet N, de Feyter PJ, Krestin GP (2014). Stress myocardial perfusion: imaging with multidetector CT. Radiology.

[14] Danad I, Szymonifka J, Schulman-Marcus J, Min JK (2016). Static and dynamic assessment of myocardial perfusion by computed tomography. Eur Heart J Cardiovasc Imaging.

[15] Kitagawa K, Goto Y, Nakamura S (2018). Dynamic CT perfusion imaging: state of the art. Cardiovasc Imaging Asia.

[16] Ziemer BP, Hubbard L, Lipinski J, Molloi S (2015). Dynamic CT perfusion measurement in a cardiac phantom. Int J Cardiovasc Imaging.

[17] Hubbard L, Ziemer B, Lipinski J et al (2016) Functional assessment of coronary artery disease using whole-heart dynamic computed tomographic perfusion. Circ Cardiovasc Imaging 9(12):1-8 10.1161/CIRCIMAGING.116.005325 PMC5517296 27956409

[18] Hubbard L, Lipinski J, Ziemer B (2018). Comprehensive assessment of coronary artery disease by using first-pass analysis dynamic CT perfusion: validation in a swine model. Radiology.

[19] Masuda T, Funama Y, Imada N (2014). Prediction of aortic enhancement on coronary CTA images using a test bolus of diluted contrast material. Acad Radiol.

[20] Hubbard L, Malkasian S, Zhao Y, Abbona P, Molloi S (2018) Contrast-to-noise ratio optimization in coronary computed tomography angiography: validation in a swine model. Acad Radiol. PMID: 30172714. 10.1016/j.acra.2018.06.026 10.1016/j.acra.2018.06.026 PMC6660910 30172714

[21] Molloi S, Zhou Y, Kassab GS (2004). Regional volumetric coronary blood flow measurement by digital angiography: in vivo validation. Acad Radiol.

[22] Garcia P, Genin G, Bret PM, Bonaldi VM, Reinhold C, Atri M (1999). Hepatic CT enhancement: effect of the rate and volume of contrast medium injection in an animal model. Abdom Imaging.

[23] Han JK, Kim AY, Lee KY (2000). Factors influencing vascular and hepatic enhancement at CT: experimental study on injection protocol using a canine model. J Comput Assist Tomogr.

[24] Di Cesare E, Gennarelli A, Di Sibio A (2014). Assessment of dose exposure and image quality in coronary angiography performed by 640-slice CT: a comparison between adaptive iterative and filtered back-projection algorithm by propensity analysis. Radiol Med.

[25] Boone J, Strauss K, Cody D (2011). Size-specific dose estimates (SSDE) in pediatric and adult body CT examinations: report of AAPM task group 204. American Association of Physicists in Medicine.

[26] Lin LI (1989). A concordance correlation coefficient to evaluate reproducibility. Biometrics.

[27] Bae KT, Heiken JP, Brink JA (1998). Aortic and hepatic contrast medium enhancement at CT. Part II. Effect of reduced cardiac output in a porcine model. Radiology.

[28] Pelgrim GJ, Nieuwenhuis ER, Duguay TM (2017). Optimal timing of image acquisition for arterial first pass CT myocardial perfusion imaging. Eur J Radiol.

[29] van Assen M, Pelgrim GJ, Slager E et al (2018) Low CT temporal sampling rates result in a substantial underestimation of myocardial blood flow measurements. Int J Cardiovasc Imaging. 10.1007/s10554-018-1451-9 10.1007/s10554-018-1451-9 PMC6454077 30284642

